# Multifeature Quantification of Nuclear Properties from Images of H&E-Stained Biopsy Material for Investigating Changes in Nuclear Structure with Advancing CIN Grade

**DOI:** 10.1155/2018/6358189

**Published:** 2018-07-05

**Authors:** Christos Konstandinou, Dimitris Glotsos, Spiros Kostopoulos, Ioannis Kalatzis, Panagiota Ravazoula, George Michail, Eleftherios Lavdas, Dionisis Cavouras, George Sakellaropoulos

**Affiliations:** ^1^Department of Medical Physics, University of Patras, Rio, Patras, Greece; ^2^Medical Image and Signal Processing Laboratory, Department of Biomedical Engineering, Technological Educational Institute of Athens, Athens, Greece; ^3^Department of Pathology, University Hospital of Patras, Rio, Greece; ^4^Department of Obstetrics and Gynecology, University Hospital of Patras, Rio, Greece; ^5^Department of Radiology and Radiotherapy, Technological Educational Institute of Athens, Athens, Greece

## Abstract

**Background:**

Cervical dysplasia is a precancerous condition, and if left untreated, it may lead to cervical cancer, which is the second most common cancer in women. The purpose of this study was to investigate differences in nuclear properties of the H&E-stained biopsy material between low CIN and high CIN cases and associate those properties with the CIN grade.

**Methods:**

The clinical material comprised hematoxylin and eosin- (H&E-) stained biopsy specimens from lesions of 44 patients diagnosed with cervical intraepithelial neoplasia (CIN). Four or five nonoverlapping microscopy images were digitized from each patient's H&E specimens, from regions indicated by the expert physician. Sixty-three textural and morphological nuclear features were generated for each patient's images. The Wilcoxon statistical test and the point biserial correlation were used to estimate each feature's discriminatory power between low CIN and high CIN cases and its correlation with the advancing CIN grade, respectively.

**Results:**

Statistical analysis showed 19 features that quantify nuclear shape, size, and texture and sustain statistically significant differences between low CIN and high CIN cases. These findings revealed that nuclei in high CIN cases, as compared to nuclei in low CIN cases, have more irregular shape, are larger in size, are coarser in texture, contain higher edges, have higher local contrast, are more inhomogeneous, and comprise structures of different intensities.

**Conclusion:**

A systematic statistical analysis of nucleus features, quantified from the H&E-stained biopsy material, showed that there are significant differences in the shape, size, and texture of nuclei between low CIN and high CIN cases.

## 1. Introduction

Cervical dysplasia concerns abnormal alterations to the cells of the cervix epithelium mainly caused by the human papillomavirus (HPV). Cervical dysplasia is a precancerous condition, and if left untreated, it may lead to cervical cancer, which is the second most common cancer in women [1]. Early diagnosis is important, since most patients can be cured if they receive early treatment. Diagnosis may be performed by a number of methods such as the Pap test, colposcopy, and histopathology. The Pap test and colposcopy have low sensitivity, and histopathology is considered the gold standard method for final diagnosis. Diagnosis of cervical dysplasia by histopathology methods comprises analysis of the suitably prepared and stained biopsy material collected from the squamous epithelium region of the cervix. Histopathology examination aims at observing, under the microscope, the existence of dysplastic or atypical immature cells in the epithelium and evaluating the extent of the epithelium covered by those cells. When abnormal cells spread into the bottom layer (basal layer) of the epithelium, the biopsy material is graded as cervical intraepithelial neoplasia (CIN) grade I, and it is regarded as mild dysplasia [2]. When abnormal cells extend into the basal and intermediate layers, the biopsy material is categorized as CIN grade II, and it is considered as moderate dysplasia. When dysplastic cells occupy the whole of the epithelium (i.e., basal, intermediate, and superficial layers), the diagnosis is CIN grade III, and it constitutes severe dysplasia. Finally, when dysplastic cells expand beyond the epithelium to surrounding tissue, it is indicative of invasive cancer [3]. Additionally, physicians assess the grade of CIN lesions by observing on histology the biopsy material nuclear parameters such as size, shape, staining, pleomorphism (variations in size, shape, and staining of nuclei), chromatin patterns, mitotic activity and mitotic figures, and presence of koilocytes and nucleoli [4, 5]. However, as shown in previous studies [6, 7], those criteria are assessed visually and are, thus, subjective leading to inter- and intrapathologists' variation as to the final diagnosis. In [7], the authors showed that agreement between 1st and 2nd readings by a panel of seven histopathologists was 65.57% regarding 6 categories: normal squamous epithelium, reactive squamous proliferation, CIN I, CIN II, CIN III, and others. In [6], the authors employed the Bethesda reporting system for assessing observer reporting variability. The Bethesda system was developed in 1998 for reporting preinvasive cervical squamous intraepithelial lesions (SILs) as of low or high grade (LSIL and HSIL). Results in [6] revealed fair inter- and intraobserver agreement.

The discrimination between low- and high-grade CIN cases is very important since low-grade cases are treated differently than high-grade cases. In particular, low-grade CIN cases are usually reversible if treated properly, but high-grade CIN cases are evolving lesions that might need surgical intervention. Failure in distinguishing the CIN grade might endanger treatment's overall efficacy [8].

To assist the diagnosis of preinvasive lesions of the cervix, a number of computer-assisted decision support systems (DSSs) have been developed. Several of those studies have employed the biopsy material stained with H&E [1, 2, 4, 5, 9–11] or Feulgen stain [12, 13] for designing DSSs. They did so by quantifying features from digital microscopy images and employing classification schemes. Those DSSs were used for discriminating between CIN grades and/or normal and malignant cervix lesions. Other studies have designed DSSs employing Pap smear images [14–16], HPV-related biomarkers [17], cervigram images [18], and clinicopathological materials [19].

In the process of designing such DSSs, a few of those studies have analyzed the discriminatory power of individual features by employing simple statistical tests such as *t*-test and ANOVA to describe changes in nuclei with advancing CIN. Huang et al. [15] and Chen et al. [16] have analyzed cell images from Pap smears and have found that dysplastic cells differ from normal cells in size, nuclear proportion, nuclear shape irregularity, chromatin density, and nuclear coarseness. Rahmadwati et al. [9] have analyzed histopathology images of the biopsy material of the cervix. They have found statistical significant differences between normal and abnormal cells, regarding 4 nucleus features (N/C, shape factor, compactness, and diameter). Sedivy et al. [5] have shown that the nuclear fractal dimension feature, which evaluates nuclear irregularity, quantified from histopathology images of the cervix, sustains statistically significant differences with the advancing CIN grade. Since nuclear atypia is considered by physicians an important parameter in assessing the CIN grade on the histopathology material, the present study is focused on quantifying nuclear atypia by analyzing in a systematic way the changes occurring in the shape, size, and texture of nuclei with the advancing CIN grade.

The contribution of the present work is as follows: (i) a number of features, regarding shape, size, and texture of nuclei, are quantified from digital images of the H&E-stained and histologically verified material; (ii) feature quantification is case centered; that is, for each patient, nuclear features are computed from the segmented nuclei of 4 or 5 regions of interest (ROIs) that have been selected by the expert physician (PR) to facilitate diagnosis; (iii) a systematic statistical analysis has been conducted for identifying features with statistical significant differences between low and high CIN cases and good correlation with the advancing CIN grade; and (iv) detailed analysis has been conducted for associating each significant feature and alteration to nuclear size, shape, and texture with the advancing CIN grade.

## 2. Materials and Methods

### 2.1. Clinical Material

The biopsy material of forty-four patients with diagnosed cervical intraepithelial neoplasia (CIN) was selected by an experienced histopathologist (PR) from the archives of the Department of Pathology, University Hospital of Patras, Rio, Greece (Table 1). The patients comprised young women from 18 to 34 years. Twenty-two of the patients had been diagnosed with low-grade squamous intraepithelial lesions (low-grade CIN) and twenty-two with high-grade squamous intraepithelial lesions (high-grade CIN).

**Figure 1:**
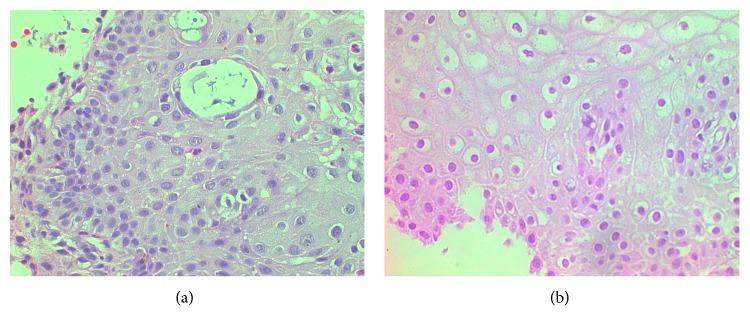
Image samples from low (a) and high (b) CIN cases.

Biopsy sections were formalin fixed, paraffin embedded, and hematoxylin and eosin (H&E) stained for histological grading. Each case was examined thoroughly under the microscope by the histopathologist, who outlined on the substrates regions where the cervix abnormalities were more obvious and appropriate for further processing. From each sample, four or five nonoverlapping images were digitized, using a digital light microscopy imaging system, comprising a Leica DM2500 light microscope equipped with a Leica DFC420C digital camera, connected to a PC, with an image resolution of 1728 × 1296 × 24 bits and TIFF file format. Images were captured at a magnification of 400x. The imaging software regulated automatically the imaging capture parameters, such as exposure time, image contrast, image amplification, gamma value, and white balance.  Figure 1 presents sample images from low and high CIN cases, respectively.

The study was conducted in accordance with the guidelines of the Declaration of Helsinki and of the Ethics Committee of the University of Patras, Greece. The study did not include live subjects, and the archive material was utilized. Informed consent was obtained from participants.

### 2.2. System Design

**Figure 2:**
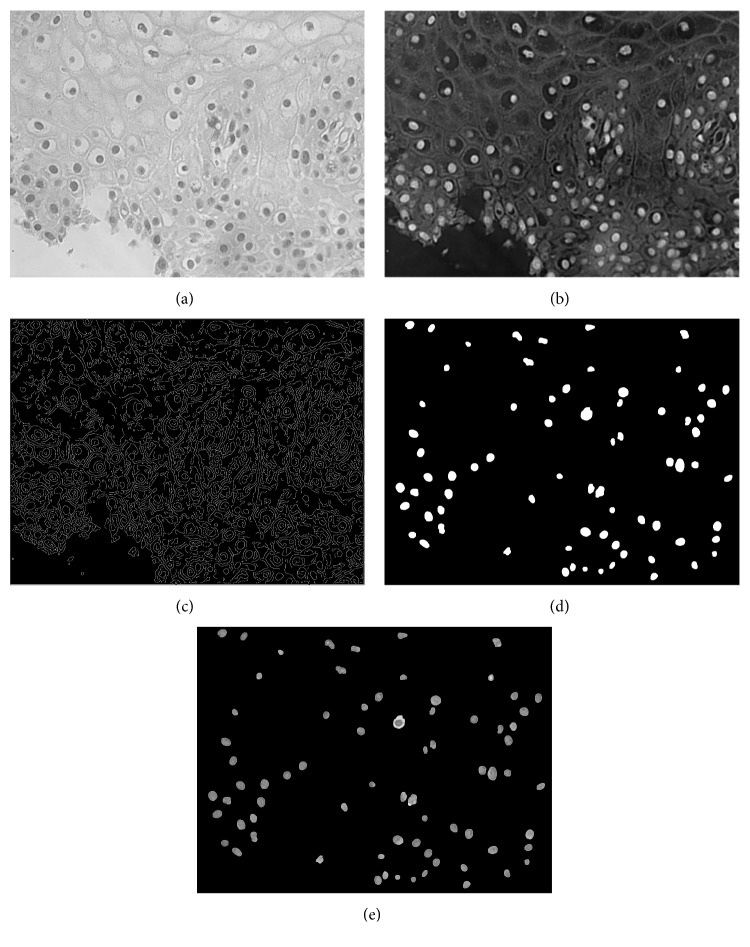
Image segmentation process: (a) grayscale image, (b) grayscale image processed by the Gaussian Laplacian filter, (c) grayscale image processed by the Canny operator, (d) grayscale image processed by morphological and size filters, and (e) final segmented image, produced by logical AND operation between (a) image and (d) image.

Images were first processed by a segmentation technique for locating the nuclei in the image. The segmentation method has been previously described in [20]. In brief, the RGB image (Figure 1(b)) was first transformed into the grayscale image (Figure 2(a)), and it was then processed by a Laplacian of Gaussian filter, which has a smoothing effect on the image (Figure 2(b)); the Canny edge detection algorithm was next employed for isolating the edges of the objects on the image (Figure 2(c)), and the resulting binary image was processed by morphological and size filters (Figure 2(d)) to complete the outline of the nuclei and to discard formations less than a preset size threshold. The latter was experimentally set to 500 pixels for the specific image resolution used in the present study. Finally, the resulting image, which was binary (Figure 2(d)), was combined by means of logical AND operation with the grayscaled image (Figure 2(a)) in order to produce the final image that contains mostly the segmented nuclei (Figure 2(e)).

The evaluation of the segmentation algorithm was performed with custom-made software specifically designed to be used by the expert physician. Accordingly, selected images from each patient and their corresponding segmented versions were displayed side by side. The expert's task was to pinpoint items on the segmented images that constituted nuclei. This procedure was repeated for all images, and the number of indicated nuclei against the total number of objects present in the segmented images provided the accuracy of the segmentation algorithm. The false-positive rate was 2%.

The next step of the computer analysis comprised the evaluation of sixty-three features from each segmented nucleus in each of the patient's images. Thus, each segmented nucleus was represented by a 63-feature vector that contained the values of the computed features. Then, a means feature vector was formed from the feature averages of all nuclei, providing a 63-feature vector that represented each patient. Feature vectors were, then, grouped into two classes, low CIN and high CIN, containing feature vectors from the corresponding low CIN and high CIN cases, respectively.

Textural features were generated from each nucleus' segmented image (such as in Figure 2(e)). Four features were computed from the nucleus histogram (mean value, standard deviation, skewness, and kurtosis). Thirteen features were calculated from the nucleus image co-occurrence matrix [21], which was computed for four directions (0°, 45°, 90°, and 135°) with the interpixel distance equal to 1. Five features were generated from the nucleus image run-length matrix [22], which was computed for four directions (0°, 45°, 90°, and 135°). Twenty-four features were computed from the discrete wavelet transform 2nd level coefficient matrices [23] along the horizontal, diagonal, and vertical directions, and eight features were computed along each direction: mean, median, maximum, minimum, range of values, standard deviation, median absolute deviation, and mean absolute deviation. Six Tamura features [24] (Tamura coarseness 1, 2, 3, and 4, contrast, and roughness) and two local binary pattern features [18] (LBP mean and standard deviation) were, also, evaluated. Morphology features, expressing size and shape nuclear attributes, were generated from the outline and area of each nucleus. Nine morphology features were calculated: six from the size of the nucleus (area, perimeter, equivalent diameter, convex area, length of the major axis, and length of the minor axis) and three from the shape of the nucleus (eccentricity, solidity, and extent). Thus, a total of 63 features were calculated from each nucleus. The 63-feature means of all nuclei from each patient's ROI images formed the 63-feature vector that represented each patient, of the verified CIN grade, for further analysis.

All the mathematical equations and the definitions of all adjustable parameters for the calculation of the abovementioned 63 features are presented at the end of this manuscript in Table 2.

The third stage of the computer analysis consisted of determining textural features sustaining statistically significant differences (SSDs) between low and high CIN cases, by means of the Wilcoxon statistical test [25], and each feature's correlation with CIN grade advancement from low to high CIN was estimated. This was expected to produce useful information regarding the variation of nucleus texture and morphology with the advancing CIN grade. The variation of feature values with the increasing CIN grade, from low to high CIN, was evaluated employing the point biserial correlation (feature values against distinct grades). The Benjamini and Hochberg FDR method was used for correcting *p* values accounting for multiple tests [26].

The proposed method was implemented in the MATLAB environment.

## 3. Results

In the image processing stage, successful identification of nuclei was achieved with an average accuracy of 89%, which is within the range of similar segmentation findings reported by previous studies [16, 27–30].

On comparing the two classes by the Wilcoxon statistical test, it was found that nuclei in low CIN and high CIN images differed in twenty-two features at the 5% (*p* < 0.05) statistical level (Table 3). After applying the Benjamini and Hochberg FDR method, 19 features from Table 3 retained statistical significance at *p* < 0.05. These features express properties related to nucleus shape and texture.

**Figure 3:**
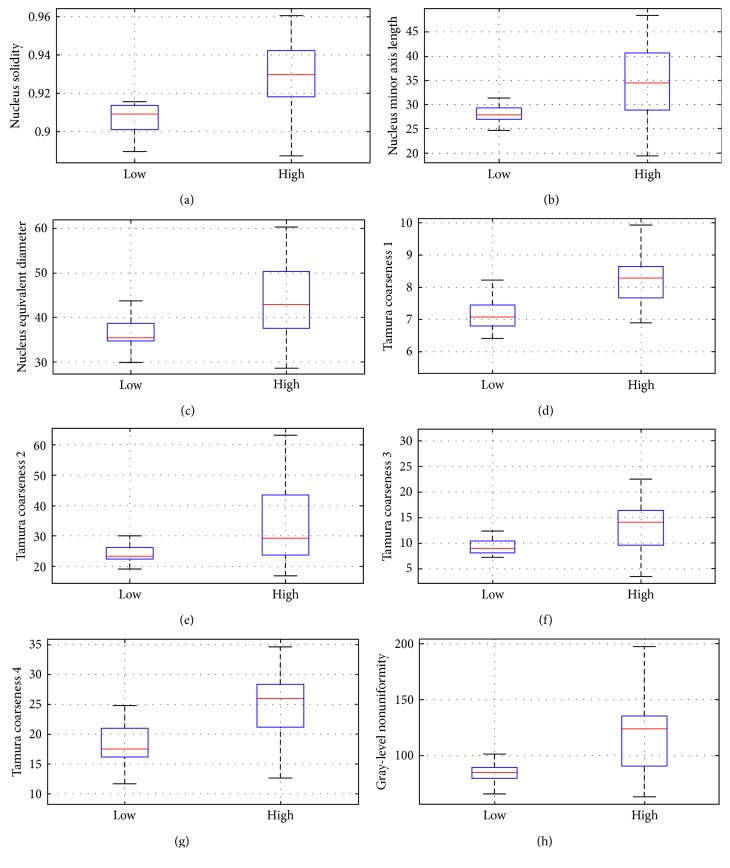
Box plots of features with high statistical significant differences and good correlation with the advancing CIN grade (*p* < 0.005, *p* corrected <0.05, and |*r*| > 0.4): (a) nucleus solidity; (b) nucleus minor axis length; (c) nucleus equivalent diameter; Tamura coarseness (d) 1, (e) 2, (f) 3, and (g) 4; (h) gray-level nonuniformity.

In particular, features of highest between-class statistical differences at the 5% level (*p* < 0.005 and *p* corrected <0.05) and of good correlation (*r* > |0.4|) with the advancing CIN grade were found in eight features: three morphological features (nucleus solidity, nucleus minor axis length, and nucleus equivalent diameter) and five textural features (4 Tamura coarseness and gray-level nonuniformity). Figures 3(a)–3(f) present the box plots of the most statistically significant features. The box plot is a graphical representation method that presents data based on their quartiles. The “box” illustrates the range of values within 25%–75% of all measurements obtained for this particular feature. The top and bottom lines depict the maximum and minimum values of all measurements obtained for this particular feature.

### 3.1. Morphological Features

Figure 3(a) shows the box plot diagram of the feature solidity (nucleus solidity), which reflects nucleus shape irregularity. Nuclei in high CIN cases displayed significantly higher border irregularities than nuclei in low CIN cases. Additionally, nucleus solidity displayed the highest correlation (*r* > 0.5) with the advancing CIN grade, as it may be verified by comparative examination of the correlation of *r* values of all features in Table 3. This is promising, since it signifies a property that perhaps could be used for establishing a segregating threshold between the two classes. Obviously, the further apart the two classes situated in the feature space, the highest the probability that such a threshold could be realistically determined.

The next two morphological features are related to the size of the nucleus, the nucleus minor axis length (nucleus minor axis length), and the nucleus equivalent diameter (nucleus equivalent diameter). Results obtained are shown in Table 3 and Figures 3(b) and 3(c). High CIN cases had nuclei significantly larger in size than nuclei in low CIN cases, having a longer minor axis length and a larger nucleus equivalent area as shown by the higher medians and spreads in Figures 3(b) and 3(c), as well as by the higher mean and standard deviation values shown in Table 3.

With regard to the rest of the morphological features that sustained statistically significant differences between low CIN and high CIN classes (nucleus area, nucleus convex area, nucleus major axis length, and nucleus perimeter), it was found that, in high CIN cases, nuclei were larger in size and in the spread of the feature values (Table 3).

Most morphological features also displayed good correlations (*r* > |0.3|) with the advancing CIN grade. Existing statistically significant differences between the two classes and good correlations of morphological features with the progression of the CIN grade indicate that there are changes occurring in the shape and size of the nuclei as the disease progresses from low CIN to high CIN.

### 3.2. Textural Features

Four Tamura coarseness features, which evaluate the coarseness of the nucleus texture, displayed high significant differences between low CIN and high CIN and very good positive correlations (*r* ≥ 0.45) with the advancing CIN grade. Feature values in high CIN cases were higher and more spread, as shown in the box plots of Figures 3(d)–3(g) and in the mean values and standard deviations of Table 3.

The gray-level nonuniformity feature (gray-level nonuniformity), which is a measure of nonuniformity in gray-level structures within the nucleus, displayed a high statistical significance difference between the two classes and the second highest ranked correlation (*r* > 0.5). The high CIN cases displayed higher median values (red line in the Figure 3(h)) and higher variances (as indicated by the spread of the corresponding box plots). This may also be verified by the corresponding data in Table 3, from where the mean value and standard deviation of the low CIN cases are significantly lower than those of the high CIN cases.

Kurtosis, which evaluates the distribution of gray-level values about the mean gray level of the nucleus, sustained high statistically significant differences between the two classes and a good positive correlation with the advancing CIN grade. High CIN cases had higher feature values and were more spread, as shown in the mean values and standard deviations of Table 3.

Three two-dimensional discrete wavelet transform features (dwt2H Mean Value, dwt2H Median Value, and dwt2H Median Absolute Deviation from the 2nd level 2D horizontal wavelet coefficient matrix) were found to sustain statistically significant differences between low CIN and high CIN cases (Table 3). The mean (dwt2H Mean Value) and median (dwt2H Median Value) features, which evaluate image coarseness in the horizontal direction, displayed statistically significant differences between the two classes. Both features displayed higher values in high CIN cases and had positive correlations, and feature values were more spread, as seen by the standard deviations in Table 3. The rest of the discrete wavelet transform features (dwt2H Mean Absolute Deviation and dwt2H Median Absolute Deviation), evaluating deviations of feature values from the median or mean values, displayed negative correlations (i.e., feature values decreased from low CIN to high CIN cases) and higher spreads, as it may be observed by the means and standard deviations of Table 3.

Two of the features emanating from the local binary pattern of the nucleus texture and evaluating the image contrast, mean (local binary pattern mean value), and standard deviation (local binary pattern standard deviation) were found to sustain statistically significant differences between the two classes and displayed positive correlations with the advancing CIN grade (*r* > 0.3) and feature values larger and more spread in the high CIN cases (Table 3).

It is also worth noticing that all features in Table 3 displayed higher spread of values in the high CIN cases, as it may be observed in the standard deviations columns.

## 4. Discussion

The material of the present study consisted of forty-four CIN cases that had been graded into two categories, low (22) and high (22) CIN cases, by an experienced pathologist. Four or five digital images per case were used, which had been previously selected by the physician, employing a microscope connected to a digital photography camera and a desktop computer. For the purpose of the present study, a custom-made software was designed that located the nuclei in all the images of each patient. Sixty-three features were calculated from each nucleus, and the means feature vector, comprising the means of all nuclei in a case, was computed to represent each particular CIN case.

Regarding nuclear features, those that revealed highest statistical significant differences (SSDs) and good correlation with the advancing CIN grade (*r* > |0.4|) were three morphological features (nucleus solidity, nucleus minor axis length, and nucleus equivalent diameter), which quantify nuclear shape and size, and five textural features (4 Tamura coarseness and gray-level nonuniformity).

Regarding morphological features with highest SSDs, the nucleus solidity feature, which estimates the nucleus shape, is quantified by the quotient of the nucleus area divided by the area of the smallest-sized convex hull polygon that can encompass the nucleus. The value of the feature increases with increasing nucleus border irregularity. As it may be observed from Figure 3(a), feature values of nuclei in high CIN cases were (a) significantly higher and (b) with larger spread amongst cases. These two findings indicate that nuclei in high CIN cases attain different and irregular shapes, and these parameters also vary significantly amongst high CIN cases. Shape irregularity has been also reported in [15, 16] (analyzing Pap smears) and [9] (quantifying nuclear features from histopathology images), as well as in [5]. Increased nuclear shape irregularity and great variation in nucleus irregularity amongst high CIN cases found in the present study reflect the fact that, in high CIN cases, nuclear atypia is dominant.

The morphological features nucleus minor axis length and nucleus equivalent diameter (the diameter of a circle with the same area as the nucleus), both related to the size of the nuclei, sustained SSDs between the two classes and positive correlations (*r* > 0.4) with the advancing CIN grade. Additionally, nuclei in high CIN cases displayed higher spreads (Figures 3(b) and 2(c)) and standard deviations (Table 3). These findings are in line with nucleus enlargement in atypia and variation in the degree of atypia, both prevailing in high CIN cases. The previous studies [15, 16] on Pap smears and the study [9] on histopathology images have also found increases in nuclear size in high CIN cases.

There were four more morphological features that sustained SSDs between the two classes: at the 1% level and correlation *r* > 0.45, the nucleus area and nucleus convex area features, and at the 5% level, the nucleus perimeter (*r* > 0.4) and nucleus major axis length. Additionally, nuclei in high CIN cases displayed higher spreads and standard deviations (Table 3) in these four features. These findings are in line with nucleus enlargement in atypia and variation in the degree of atypia which prevail in high CIN cases.

With regard to textural features, four Tamura coarseness features were found to sustain SSDs between high CIN and low CIN cases at the 5% level and displayed positive correlations (*r* > |0.4|). This becomes evident from Table 3, where mean values and standard deviations are higher in the high CIN cases, and Figures 3(d)–3(g), where the medians and spreads in the box plots are higher in the high CIN cases. These findings indicate that nuclei in high CIN cases appear coarser; that is, the nucleus texture contains smaller numbers of large primitives or texture elements (texels), and that image coarseness varies more amongst high CIN cases. This is probably related to the predominance of atypical nuclei in high CIN cases. Higher nuclear coarseness in high CIN cases has been also reported in [15, 16] on Pap smears.

Two more textural features, kurtosis and gray-level nonuniformity, sustained high SSDs between high CIN and low CIN cases and displayed positive correlations (*r* = 0.35 and *r* = 0.52, resp.) with the advancing CIN grade (Table 3). Kurtosis is related to the distribution of graytone intensities on the nucleus texture, and gray-level nonuniformity is a measure of nonuniformity in gray-level structures that comprise the nucleus texture. These findings indicate that nucleus texture in high CIN cases contains structures of different gray-level intensities and that these distributions vary amongst cases.

The “horizontal detail” (H) discrete wavelet transform features were found to sustain SSDs between high CIN and low CIN cases. Positive correlation with the advancing CIN grade (*r* > 0.3) displayed those dwt2 features that evaluate the mean and median of the H-image (dwt2H Mean Value and dwt2H Median Value), and negative correlations displayed those that evaluate the mean and the median of the absolute deviation (dwt2H Mean Absolute Deviation and dwt2H Median Absolute Deviation) (Table 3). These findings indicate that, in the case of the median and mean value dwt2 features (dwt2H Mean Value and dwt2H Median Value), the nuclei in high CIN cases had larger magnitude edges as compared to nuclei in low CIN cases. This may be attributed to the hyperchromasia or higher staining than normal of nuclei with atypia, which prevails in high CIN cases. The lower values in high CIN cases in the dwt2 features that evaluate deviation (dwt2H Mean Absolute Deviation and dwt2H Median Absolute Deviation) indicate consistency in the size of edges amongst high CIN cases.

Finally, two more textural features, local binary pattern mean value and local binary pattern standard deviation, sustained SSDs between high and low CIN cases. The LBP quantifies the local contrast of the nucleus texture. The features local binary pattern mean value and local binary pattern standard deviation quantify the mean and the variation of the local contrasts on the nucleus texture. It was found that both features had larger values in high CIN cases, which indicates that the textural contrast of nuclei is higher with higher variation amongst high CIN cases.

Several of the above morphological and textural features quantify similar properties of the nuclei, such as the nucleus image structure, size, and shape. Nevertheless, those features had to be examined, with regard to the particular property they quantify, for reassuring findings as to how particular nucleus properties change with the advancing CIN grade. This is probably connected to higher nuclear atypia and variation in the degree of nuclear atypia which prevail in high CIN cases.

Summarizing, this study showed that nuclei in high CIN cases, in comparison to nuclei in low CIN cases, attain more irregular shape and are larger in size, and the nucleus texture becomes coarser, contains higher edges, is of higher local contrast, is more inhomogeneous, and contains structures of different intensities. These properties seem to vary a lot in the nuclei of high CIN cases, except for the existence of high edges on the nucleus surface.

## Figures and Tables

**Table 1:** Data clinical annotations.

Categorization	Biopsy (CIN diagnosis)			Pap test diagnosis				
	CIN I	CIN II	CIN II-III	Normal	HPV	ASCUS	CIN I	CIN II-III
Low grade	22	—	—	1	3	9	9	—
High grade	—	15	7	—	1	5	11	5

**Table 2:** 

	(*1) Histogram features*	
1	Mean value	*m*=∑_*i*_∑_*j*_ *g*(*i*, *j*)/*N*, where *g*(*i*, *j*) is the pixel intensity in position (*i*, *j*) and *N* is the total number of pixels
2	Standard deviation	$\text{std}=\sqrt{\sum_{i}\sum_{j}\;{\left(g\left(i,j\right)-m\right)}^{2}/N}$
3	Skewness	sk=(1/*N*)(∑_*i*_∑_*j*_ (*g*(*i*, *j*) − *m*)^3^/std^3^)
4	Kurtosis	*k*=(1/*N*)(∑_*i*_∑_*j*_ (*g*(*i*, *j*) − *m*)^4^/std^4^)

	*(2) Co-occurrence matrix-based features*	
5	Angular second moment	ASM=∑_*i*=0_^*N*_g_−1^∑_*j*=0_^*N*_g_−1^(*p*(*i*, *j*))^2^, where *N*_g_ is the number of gray levels in the image, *i*, *j*=1,…, *N*_g_, and *p*(*i*, *j*) is the co-occurrence matrix. ASM describes image smoothness and takes minimum values for smooth-textured nuclei. *p*(*i*, *j*) was calculated using the MATLAB function *graycomatrix*
6	Contrast	CON=∑_*n*=0_^*N*_g_−1^ *n*^2^{∑_*i*=0_^*N*_g_−1^∑_*j*=0_^*N*_g_−1^(*p*(*i*, *j*))^2^}, |*i* − *j*|=*n*
7	Inverse different moment	IDM=∑_*i*=0_^*N*_g_−1^∑_*j*=0_^*N*_g_−1^ *p*(*i*, *j*)/1+(*i* − *j*)^2^
8	Entropy	ENT=−∑_*i*=0_^*N*_g_−1^∑_*j*=0_^*N*_g_−1^ *p*(*i*, *j*)log(*p*(*i*, *j*))
9	Correlation	COR=∑_*i*=0_^*N*_g_−1^∑_*j*=0_^*N*_g_−1^(*ij*)*p*(*i*, *j*) − *m*_*x*_*m*_*y*_/*σ*_*x*_*σ*_*y*_, where *m*_*x*_, *m*_*y*_, *σ*_*x*_, and *σ*_*y*_ are the respective mean values and standard deviations of *p*_*x*_ and *p*_*y*_, described below: *p*_*x*_(*i*)=∑_*j*=1_^*N*_rows_^ *p*(*i*, *j*) *p*_*y*_(*j*)=∑_*j*=1_^*N*_columns_^ *p*(*i*, *j*)
10	Sum of squares	SSQ=∑_*i*=0_^*N*_g_−1^∑_*j*=0_^*N*_g_−1^(1 − *m*)^2^*p*(*i*, *j*)
11	Sum average	SAVE=∑_*i*=2_^2*N*_g_^ *ip*_*x*+*y*_(*i*), where *p*_*x*+*y*_ is *p*_*x*+*y*_(*k*)=∑_*i*=1_^*N*_g_^∑_*j*=1_^*N*_g_^ *p*(*i*, *j*), *i*+*j*=*k*, *k*=2,3,…, 2*N*_g_
12	Sum entropy	SENT=−∑_*i*=2_^2*N*_g_^ *p*_*x*+*y*_(*i*)log(*p*_*x*+*y*_(*i*))
13	Sum variance	SVAR=−∑_*i*=2_^2*N*_g_^ (*i* − SENT)^2^*p*_*x*+*y*_(*i*)
14	Difference variance	DVAR=∑_*i*=2_^2*N*_g_^ (*i* − SAVE)^2^*p*_*x*−*y*_(*i*)
15	Difference entropy	DENT=−∑_*i*=0_^*N*_g_−1^*p*_*x*−*y*_(*i*)log(*p*_*x*−*y*_(*i*)), where *p*_*x*+*y*_ is *p*_*x*−*y*_(*k*)=∑_*i*=1_^*N*_g_^ ∑_*j*=1_^*N*_g_^ *p*(*i*, *j*), |*i* − *j*|=*k*, *k*=2,3,…, *N*_g_ − 1
16	Information measure of correlation 1	ICM1=*HXY* − *HXY*1/max{*HX*, *HY*}
17	Information measure of correlation 2	ICM2=(1 − exp[−2.0(*HXY*2 − *HXY*)])^1/2^, where *HXY*=−∑_*i*=0_^*N*_g_−1^∑_*j*=0_^*N*_g_−1^*p*(*i*, *j*)log(*p*(*i*, *j*)), *HXY*1=−∑_*i*=0_^*N*_g_−1^∑_*j*=0_^*N*_g_−1^*p*(*i*, *j*)log(*p*_*x*_(*i*)*p*_*y*_(*j*)), *HXY*2=−∑_*i*=0_^*N*_g_−1^∑_*j*=0_^*N*_g_−1^*p*_*x*_(*i*)*p*_*y*_(*j*)log(*p*_*x*_(*i*)*p*_*y*_(*j*))

	*(3) Run-length matrix-based features*	
18	Short-run emphasis	SRE=∑_*i*_^*N*_g_^∑_*j*_^*N*_r_^ *Q*_RL_(*i*, *j*)/*j*^2^/∑_*i*_^*N*_g_^∑_*j*_^*N*_r_^ *Q*_RL_(*i*, *j*), where *Q*_RL_(*i*, *j*) is the run-length matrix, *N*_g_ is the number of gray values in the image, *N*_r_ is the largest possible run, *i*=1,…, *N*_g_, and *j*=1,…, *N*_r_
19	Long-run emphasis	LRE=∑_*i*_^*N*_g_^∑_*j*_^*N*_r_^ *Q*_RL_(*i*, *j*) · *j*^2^/∑_*i*_^*N*_g_^∑_*j*_^*N*_r_^ *Q*_RL_(*i*, *j*)
20	Gray-level nonuniformity	GLNU=∑_*i*_^*N*_g_^[∑_*j*_^*N*_r_^ *Q*_RL_(*i*, *j*)]^2^/∑_*i*_^*N*_g_^∑_*j*_^*N*_r_^ *Q*_RL_(*i*, *j*)
21	Run-length nonuniformity	RLNU=∑_*j*_^*N*_r_^[∑_*i*_^*N*_g_^ *Q*_RL_(*i*, *j*)]^2^/∑_*i*_^*N*_g_^∑_*j*_^*N*_r_^ *Q*_RL_(*i*, *j*)
22	Run percentage	RP=∑_*i*_^*N*_g_^∑_*j*_^*N*_r_^ *Q*_RL_(*i*, *j*)/*P*, where *P* is the total number of pixels in the image
	*(4) Wavelet-based features*	
23	dwt2H Mean Value	MATLAB function: *mean(W(:))*, where *W* is the 2nd level dwt in the horizontal direction
24	dwt2H Median Value	MATLAB function: *median(W(:))*
25	dwt2H Max Value	MATLAB function: *max(W(:))*
26	dwt2H Min Value	MATLAB function: *min(W(:))*
27	dwt2H Range of Values	MATLAB function: *range(W(:))*
28	dwt2H Standard Deviation	MATLAB function: *std(W(:))*
29	dwt2H Median Absolute Deviation	MATLAB function: *mad(W(:),1)*
30	dwt2H Mean Absolute Deviation	MATLAB function: *mad(W(:),0)*
31–38	same as 23–30, where *W* is the 2nd level dwt in the diagonal direction (MATLAB function *dwt2*)	
39–46	same as 23–30, where *W* is the 2nd level dwt in the vertical direction	

	*(5) Tamura-based features*	
47	Tamura coarseness 1	*F*_crs_=(1/*m* × *n*)∑_*i*_^*m*^ ∑_*j*_^*n*^ *S*_best_(*i*, *j*), where *m* and *n* are region dimensions and *S*_best_(*i*, *j*)=2^*k*^, in which *k* is the best scaling for highest neighborhood average
48–50	Tamura coarseness 2–4	Values of the 3-bin histogram of *S*_best_
51	Tamura contrast	*F*_con_=*σ*/(*a*_4_)^*n*^, where *σ* is the standard deviation and *a*_4_ is the kurtosis
52	Tamura roughness	*F*_rgh_=*F*_crs_+*F*_con_

	*(6) Local binary pattern-based features*	
53	LBP mean	Mean value of the LBP histogram: LBP_*P*, *R*_=∑_*p*=0_^*P*−1^ *s*(*g*_p_ − *g*_c_)2^*P*^, where *g*_p_ and *g*_c_ are, respectively, gray-level values of the central pixel and *P* surrounding pixels in the circle neighborhood of radius *R* and $s\left(x\right)=\left\{\begin{array}{cc} 1, & \text{if}\;x\geq 0\\ 0, & \text{otherwise} \end{array}\right.$
54	LBP standard deviation	Standard deviation of the LBP histogram

	*(7) Morphological-based features*	
55	Nucleus area	MATLAB function: *regionprops(BW, properties)*, where *BW* is the binary image nucleus and *properties* = *‘Area'*
56	Nucleus perimeter	Where *properties* = *‘Perimeter'*
57	Nucleus equivalent diameter	Where *properties* = *‘EquivDiameter'*
58	Nucleus convex area	Where *properties* = *‘ConvexArea'*
59	Nucleus major axis length	Where *properties* = *‘MajorAxisLength'*
60	Nucleus minor axis length	Where *properties* = *‘MinorAxisLength'*
61	Nucleus eccentricity	Where *properties* = *‘Eccentricity'*
62	Nucleus solidity	Where *properties* = *‘Solidity'*
63	Nucleus extent	Where *properties* = *‘Extent'*

The parameters used for calculation of the abovementioned features were the following: (1) histogram features: the number of grayscale values = 256. (2) Co-occurrence matrix-based features: directions (0°, 45°, 90°, and 135°), interpixel distance = 1, and the number of grayscale values = 16. (3) Run-length matrix-based features: directions (0°, 45°, 90°, and 135°) and the number of grayscale values = 16. (4) Wavelet-based features: MATLAB function dwt2, Daubechies 2 transform, 2nd level coefficient matrices along the horizontal, diagonal, and vertical directions, and the number of grayscale values = 256. (5) Tamura-based features: *k* = 0 : 5. (6) Local binary pattern-based features: *R*=1 and *p*=8. (7) Morphological-based features: MATLAB function regionprops.

**Table 3:** Morphological and textural features with statistically significant differences between low and high CIN classes, ranked alphabetically.

	Feature name	*p* < 0.05	*p* corrected^*∗*^	*r* (correlation)	Low CIN		High CIN	
					Mean	SE^*∗∗*^	Mean	SE
1	dwt2D Mean Absolute Deviation	4.48*E* − 02	1.34*E* − 01	−0.34	6.23	0.13	5.50	0.28
2	dwt2H Mean Absolute Deviation	2.02*E* − 03	1.82*E* − 02	−0.39	10.44	0.24	8.67	0.59
3	dwt2H Mean Value	6.25*E* − 03	3.03*E* − 02	0.27	−0.84	0.029	−0.68	0.080
4	dwt2H Median Absolute Deviation	7.20*E* − 03	3.24*E* − 02	−0.33	1.17	0.065	0.95	0.072
5	dwt2H Median Value	5.03*E* − 05	3.17*E* − 03	0.18	−0.014	0.0024	−0.0079	0.0048
6	dwt2H Standard Deviation	2.50*E* − 02	7.87*E* − 02	−0.31	23.33	0.39	20.80	1.10
7	Gray-level nonuniformity	2.02*E* − 03	2.13*E* − 02	0.52	85.21	1.98	116.83	7.71
8	Kurtosis	2.98*E* − 03	2.35*E* − 02	0.37	2.59	0.02	2.71	0.04
9	Local binary pattern mean value	1.02*E* − 02	3.77*E* − 02	0.40	16.61	0.23	18.03	0.45
10	Local binary pattern standard deviation	1.37*E* − 04	4.30*E* − 03	−0.47	0.12	0.00082	0.12	0.0014
11	Nucleus area	7.20*E* − 03	3.02*E* − 02	0.46	1141	50	1640	137
12	Nucleus convex area	8.27*E* − 03	3.26*E* − 02	0.45	1253	53	1772	147
13	Nucleus equivalent diameter	5.03*E* − 03	2.64*E* − 02	0.47	36.53	0.78	43.59	1.85
14	Nucleus extent	5.00*E* − 02	1.43*E* − 01	0.22	0.64	0.0045	0.66	0.0079
15	Nucleus major axis length	1.16*E* − 02	4.07*E* − 02	0.37	52.6	1.7	60.1	2.3
16	Nucleus minor axis length	3.75*E* − 03	2.36*E* − 02	0.47	28.0	0.5	34.1	1.7
17	Nucleus perimeter	1.24*E* − 02	4.12*E* − 02	0.40	135.9	3.2	156.6	6.4
18	Nucleus solidity	1.37*E* − 04	2.87*E* − 03	0.52	0.91	0.0027	0.93	0.0037
19	Tamura coarseness 1	1.15*E* − 03	1.81*E* − 02	0.45	7.12	0.12	8.01	0.24
20	Tamura coarseness 2	4.04*E* − 03	2.31*E* − 02	0.48	23.88	0.57	33.99	2.77
21	Tamura coarseness 3	2.98*E* − 03	2.09*E* − 02	0.45	9.11	0.37	14.27	1.51
22	Tamura coarseness 4	1.73*E* − 03	2.18*E* − 02	0.49	18.21	0.70	23.94	1.40

^*∗*^Benjamini and Hochberg FDR correction; MATLAB function: *mafdr()*; ^*∗∗*^SE = standard error.
